# Cold sensitivity and its association to functional disability following a major nerve trunk injury in the upper extremity—A national registry-based study

**DOI:** 10.1371/journal.pone.0270059

**Published:** 2022-07-12

**Authors:** Drifa Frostadottir, Linnéa Ekman, Malin Zimmerman, Lars B. Dahlin

**Affiliations:** 1 Department of Translational Medicine–Hand Surgery, Lund University, Malmö, Sweden; 2 Department of Hand Surgery, Skåne University Hospital, Malmö, Sweden; 3 Department of Orthopedics, Helsingborg Hospital, Helsingborg, Sweden; 4 Department of Biomedical and Clinical Sciences, Linköping University, Linköping, Sweden; BG Trauma Center Ludwigshafen, GERMANY

## Abstract

**Aims:**

To investigate self-reported cold sensitivity and functional disability after a repaired major nerve trunk injury in the upper extremity.

**Methods:**

We identified 735 individuals with a major nerve trunk injury in the upper extremity, surgically treated with direct nerve repair or reconstructed with nerve autografts, in the Swedish national quality registry for hand surgery (HAKIR). Patient-reported symptoms, including cold sensitivity, and perceived disability were collected using two questionnaires (HQ-8 and QuickDASH) preoperatively, and at three and 12 months postoperatively.

**Results:**

We included 281 individuals, who had responded the questionnaires, where 197 (70%) were men (median age 34 [interquartile range 25–52] years) and 84 (30%) were women (median age 41 [25–55]). Cold sensitivity (scored 0–100) was the most prominent symptom 12 months postoperatively after an injured and repaired/reconstructed median (p<0.001) or ulnar (p<0.001) nerve, while individuals with a radial nerve injury showed milder symptoms. Concomitant injuries did not affect cold sensitivity scores. Individuals with ulnar nerve injuries scored higher in stiffness (p = 0.019), weakness (p<0.001) and ability to perform daily activities (p = 0.003) at 12 months postoperatively than median nerve injuries. Individuals with a median, ulnar or radial nerve injury with severe (>70) cold sensitivity had 25, 37 and 30 points higher QuickDASH scores, respectively (p<0.001), at 12 months postoperatively than individuals with mild (<30) cold sensitivity. There were no differences in QuickDASH score or cold sensitivity score at 12 months postoperatively between direct nerve repair or nerve reconstruction with nerve autografts. Neither age, nor sex, affected QuickDASH score at 12 months postoperatively.

**Conclusion:**

Cold sensitivity after surgery for a major nerve trunk injury in the upper extremity can be substantial with impaired ability to perform daily activities, where an ulnar nerve injury may have a worse outcome.

## Introduction

Disability after a repaired or reconstructed major nerve trunk injury in the upper extremity may be extensive. The individual´s function may not only be related to pure motor and sensory dysfunction, but also to other modalities that should be assessed by various instruments [1–3]. An important remaining symptom after a nerve injury is cold sensitivity, that can be evaluated by using different questionnaires during follow-up [4,5]. Cold sensitivity is defined by four types of symptoms on exposure to cold: 1) pain/discomfort, 2) stiffness, 3) altered sensibility and 4) color change [6] in the area innervated by the injured nerve. These symptoms can occur isolated, or in combinations. In most cases the symptoms appear within the first six months and can persist for many years [4,7,8], with the risk of becoming permanent [9]. Cold sensitivity may be associated with neuropathic pain [10] as well as with loss of function [11]. Interestingly, an improved sensory function after treatment may reduce cold sensitivity [12].

Prognostic risk factors for outcome of surgery for median and ulnar nerve injuries have been previously presented [13–16]. Recovery of sensory function, power grip and a global functional score, evaluated by the disabilities of the arm, shoulder and hand questionnaire (DASH), seem to be favourable prognostic factors after treated median and ulnar nerve injuries [14], but few studies have been dedicated to the relevance of cold sensitivity. Previous data indicate that cold sensitivity may be more frequent after an ulnar nerve injury than after a median nerve injury [17]. Outcome and presence of cold sensitivity after surgically treated hand and nerve injuries or disorders can be evaluated by patient related outcome measurements (PROMs) [18–20]. In the Swedish National Quality Register for Hand Surgery (HAKIR), symptoms and disability are evaluated pre- and postoperatively by QuickDASH and a specific hand surgery questionnaire (HQ-8), focusing on pain, cold sensitivity and related symptoms with subsequent ability to perform daily activities [5]. Data from HAKIR indicates problems with cold sensitivity even after treated single digital nerve injuries [20]. However, the presence of cold sensitivity after surgically treated major nerve trunks has not been sufficiently highlighted, nor how it is affected by concomitant injuries. It is also unknown how it is related to other modalities, as well as to function and ability to perform daily activities. Our aims were to investigate the presence of cold sensitivity in individuals surgically treated for a major nerve trunk injury affecting the median, ulnar or radial nerve and to evaluate any prognostic factors for developing cold sensitivity up to 12 months using data from a national quality registry for hand surgery.

## Materials and methods

### Ethical approval and consent to participate

This study was approved by the Regional Ethical Review Board in Stockholm, Sweden, and the national Ethical Review Board (2017/3:11; Stockholm, Sweden, and 2021–00902; Uppsala, Sweden). The research was performed according to the Helsinki Declaration. Each individual provided informed written consent before inclusion in HAKIR and NDR. Data is only presented at group level and no particular individual can be identified from the data.

### Cohort

In the present retrospective study, individuals aged 18 or above, operated with surgical repair or reconstruction with sensory nerve autografts of a single major nerve trunk injury (i.e. median, ulnar or radial nerve injury at wrist or forearm level) 2010–2018, were selected for the study from the Swedish National Quality Registry for Hand Surgery (HAKIR) [18]. Individuals were identified through ICD-10 [International Statistical Classification of Diseases and Related Health Problems [21]] diagnosis codes S640, S641, S642 S540, S541 and S542 as well as the surgical procedure codes (KKÅ97) ACB21, ACB22, ACB23 ACB29, ACC22, ACC23, and ZZK00. The exclusion criteria were surgery with nerve biopsy, combined injuries to both digital nerves and major nerve trunks or surgery for multiple nerve trunk injuries.

### Data sources

Individuals with an injury to a major nerve trunk in the upper extremity were invited once admitted to the respective hand surgery department to partake by filling in the validated Swedish versions of QuickDASH and the HAKIR Questionnaire-8 (HQ-8) at baseline (preoperatively; evaluating the symptoms and disability during the last week) and at three and 12 months postoperatively either by post or online having two weeks to answer to be included in the registry. The QuickDASH questionnaire assesses self-reported arm-function and is scored from 0–100, where a higher score represents more severe disability [22]. The validated HQ-8 questionnaire measures unique aspects of disability in the entire affected upper extremity and consists of seven questions on perceived symptoms in the affected hand and arm (i.e. pain at rest, pain at motion without load, pain at load, stiffness, weakness, numbness/tingling, cold sensitivity) and one question on the ability to perform daily activities [5]. Each symptom is graded by the individual from 0–100 on a Likert scale, with a higher score representing more severe problems [5]. Though not a disease-specific outcome instrument, the HQ-8 questionnaire complements other PROMs, such as the QuickDASH questionnaire, with symptom-related questions. While it shares some features with the validated Cold Intolerance Symptom Scale (CISS) [23,24], the HQ-8 questionnaire is simpler and considered easier to use in routine clinical practice as well as to engage a larger population to partake [23,24].

Included individuals were divided into three groups according to involved nerve in the upper extremity–the median, ulnar or radial nerve trunks. Data on any concomitant injury, such as flexor tendon injury, extensor tendon injury, fracture in the upper extremity, joint/ligament injury and vascular injury, was also retrieved from the HAKIR database. Isolated and multiple concomitant injuries were analysed and presented together. We classified cold sensitivity into three categories according to severity at 12 months postoperatively for each of the three nerve groups, as previously done [19]. Mild cold sensitivity was defined as a HQ-8 score of ≤30, moderate cold severity as a score of 31–70 and severe cold severity as a score of >70.

The HAKIR database was linked to The Swedish National Diabetes Registry (NDR) (http://www.ndr.se) through personal identifying numbers and data on the individuals’ diabetic status in our study was retrieved. NDR is a national quality register that includes all individuals above 18 years diagnosed with type 1 or 2 diabetes. Individuals with diabetes were identified and diabetes was adjusted for due to the risk for presence of pre-injury cold sensitivity among such patients [25].

### Statistics

Data are presented as median [interquartile range, IQR] or numbers (%). Fisher´s exact test was used to compare the frequency of the operated hand (left vs right hand). The Kruskal-Wallis test was used for significance testing for continuous variables, with Mann-Whitney U-test as the post-hoc test. Mann-Whitney U-test was also used for sex, age and seasonal comparisons. The Chi-Square test was used for nominal variables.

In order to predict cold sensitivity, a multiple linear regression analysis was performed with cold sensitivity at 12 months as the dependent continuous variable, adjusted for age, sex, diabetes and the most common concomitant injury for each nerve. To assess the association between cold sensitivity and functional disability, a univariate general linear analysis was performed with the QuickDASH score at 12 months as the dependent continuous variable and the three cold sensitivity categories as the independent variables. The model was adjusted for age at surgery, sex, diabetes as well the most common concomitant injury for each nerve. Values are expressed in points as unstandardized B values with 95% confidence intervals.

## Results

### Included individuals

**Fig 1:**
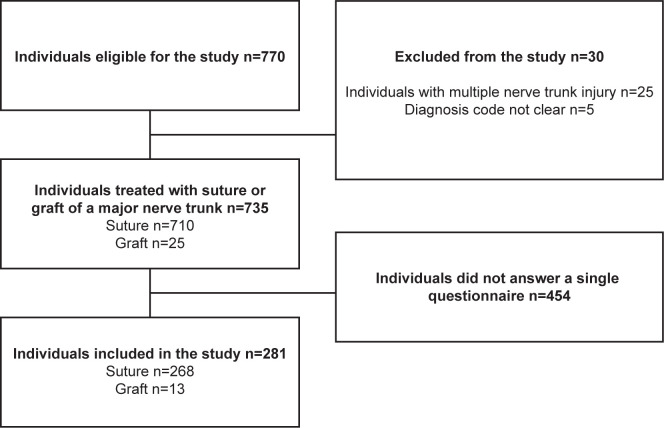
Study patient flowchart.

During the study period, 770 individuals with a single nerve trunk injury in the upper extremity (median, ulnar, or radial nerves) were identified in HAKIR. Of those individuals, 25 were treated for multiple nerve trunk injuries and ten did not have a clearly documented diagnosis code. The remaining individuals included 710 single major nerve trunk injuries treated with a direct nerve repair with sutures and 25 major nerve trunk injuries treated with reconstruction with nerve autografts. Individuals with or without concomitant injury in the hand were defined and included in the study. Of these, 268/710 (38%) individuals, treated with direct nerve repair with sutures, and 13/25 (52%) individuals, treated with nerve autografts, had responded to at least one HAKIR questionnaire at baseline and/or at three months and/or at 12 months postoperatively and were included in the study (n = 281; Fig 1).

### Baseline characteristics

Of the included individuals, there were 197/281 (70%) men and 84/281 (30%) women. No significant difference in age between men and women (median age 34 [interquartile range; IQR 25–52] years and 41 [25–55] years, respectively; p = 0.234) was observed. The left hand was more commonly injured than the right hand (145/281, 54%; p<0.001). A diagnosis of diabetes before the nerve injury was confirmed through data obtained from NDR in 9/281 (3.2%) individuals (two type 1 diabetes and seven type 2 diabetes; Table 1).

**Table 1:** Baseline characteristics of individuals treated with repair or reconstruction of a major nerve trunk injury in the upper extremity.

	Median nerve n = 115	Ulnar nerve n = 94	Radial nerve n = 72	P-value
Age at surgery (years)	35 [24–52]	37 [25–54]	38 [26–57]	0.79
Sex (male)	75 (65)	64 (68)	58 (80)	0.07
***Type of repair***				
Nerve repair with sutures	111 (97)	89 (95)	68 (94)	0.75
Autologous nerve grafting	4 (3)	5 (5)	4 (6)	
***Concomitant injuries***				
Flexor tendon injury	68 (59)	46 (49)^b^	11 (15) ^c^	**<0.001**
Extensor tendon injury	3 (3)	2 (2)^b^	30 (42) ^c^	**<0.001**
Fracture in the upper extremity	1 (1)	0 (0)	3 (4)	0.07
Joint/ligament injuries	0 (0)	0 (0)	1 (1)	0.23
Vascular injury	12 (10)^a^	28 (30)^b^	6 (8)	**<0.001**
***Season of surgery***				
Surgery during summer	70 (61)	56 (60)	42 (58)	0.94

Values are presented as n (%) or median [IQR; interquartile range]. Significance testing with Kruskal-Wallis and post-hoc Mann-Whitney U-test were used.

*Bold values indicate p <0.05 and are considered statistically significant.

*a* indicates p<0.05 between median and ulnar nerve values

*b* indicates p < 0.05 between ulnar and radial nerve values

*c* indicates p <0.05 between median and radial nerve values.

The most commonly injured major nerve trunk was the median nerve [115/281 (41%)] for both men (38%) and women (48%), followed by the ulnar nerve [94/281 (33%)] and the radial nerve [72/281 (26%)] (Table 1). A drop-out analysis of the the 454/735 individuals that had suffered a single nerve trunk injury and were not included in the study showed a similar distribution of injury for each nerve trunk, where the median nerve was the most commonly injured nerve [187/454 (41%)], followed by the ulnar nerve [153/454 (34%)] and radial nerve [114/454 (25%)]. No difference was found in the age of men or women compared to those included in the study (median age 33 [24–48] years and 38 [27–54] years; p = 0.289 and p = 0.999, respectively). Gender distribution was similar for the individuals not included in the study [men 318/454 (70%) and women 136/454 (30%)].

Of the 281 included individuals, 13 were treated with a nerve autograft for a single major nerve trunk injury (5%). None of the individuals treated with nerve autograft had diabetes at time of surgery.

### Concomitant injuries

The most frequent concomitant injury for a median or an ulnar nerve trunk injury was flexor tendon injury (at hand, wrist or forearm level), occurring in 68/115 (59%) and 46/94 (49%) of the individuals, respectively. There was no difference in cold sensitivity or in QuickDASH score at 12 months postoperatively for individuals with a median nerve injury with or without a concomitant flexor tendon injury (p = 0.417; p = 0.158; respectively) nor an ulnar nerve injury with or without concomitant flexor tendon injury (p = 0.204; p = 0.264, respectively; data not shown).

The most frequent concomitant injury for a radial nerve trunk injury was extensor tendon injury (at hand, wrist or forearm level), occurring in 30/72 (42%) of the individuals. There was no difference in cold sensitivity or in QuickDASH score at 12 months postoperatively for individuals with a radial nerve injury with or without a concomitant extensor tendon injury (p = 0.250 and p = 0.463, respectively).

Vascular injury was most frequently associated with an ulnar nerve injury, occurring in 28/94 (30%) of the individuals compared to 12/115 (10%) and 6/72 (8%) of individuals with a median or a radial nerve injury, respectively (Table 1). There was no difference in cold sensitivity or in QuickDASH score at 12 months postoperatively between individuals with an ulnar nerve injury with or without a concomitant vascular injury (p = 0.202 and p = 0.103, respectively).

### Type of nerve repair and reconstruction

Nerve repair with sutures was the most common treatment for individuals with an injury to a major nerve trunk, where nerve autografting was needed in 4/115 (3%), 5/94 (5%) and 4/72 (6%) individuals with a median, ulnar or radial nerve injury, respectively (Table 1). No difference was seen in cold sensitivity score or in QuickDASH score at 12 months for individuals with direct sutures vs. autografting of a major nerve trunk injury (p = 0.960 and p = 0.981, respectively).

### Time of surgery

During our study period, 168/281 (60%) of the surgeries were performed during the summer period (April-September) and 113/281 (40%) during the winter period (October-March) (Table 1). There was no difference in reported cold sensitivity between individuals, with any of the three nerve injuries, operated during the summer period compared to the individuals operated during the winter period at three (p = 0.305) or at 12 months (p = 0.797) postoperatively.

### Cold sensitivity with related symptoms

**Fig 2:**
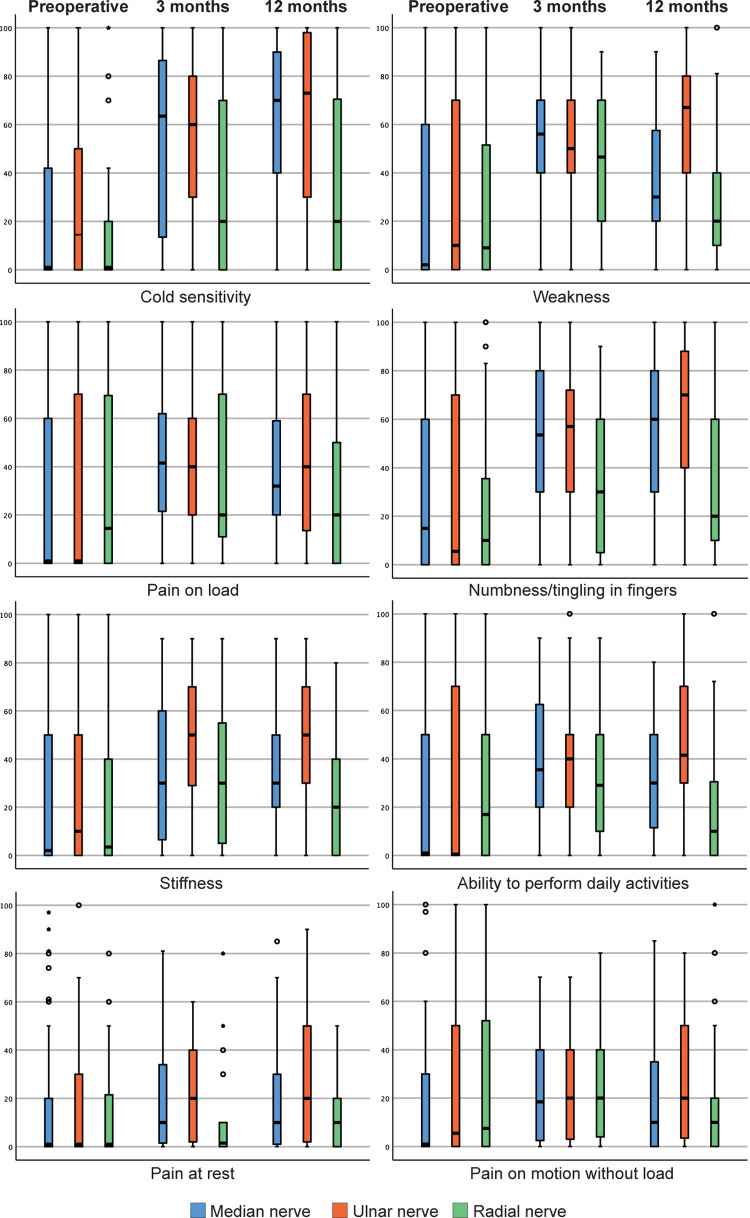
Boxplot of HAKIR questionnaire (HQ-8) results over time for patients with a median, an ulnar or a radial nerve injury. All eight questions presented individually, graded from 0–100 on a Likert scale, with a higher score representing more severe symptoms.

Individuals with a median or an ulnar nerve injury had developed cold sensitivity at three months postoperatively, which persisted at 12 months postoperatively (p<0.001 and p<0.001, respectively) (Table 2, Fig 2). The score for cold sensitivity did not differ over time for the group with a radial nerve injury. Presence of pain on load, but not for the other pain modalities, was seen for the individuals with a median, but not for an ulnar or a radial, nerve injury at three months postoperatively, which persisted at 12 months postoperatively (p<0.022).

**Table 2:** HAKIR questionnaire (HQ-8) results in 281 individuals with a median, an ulnar or a radial nerve injury treated with sutures or nerve autografting.

HQ-8 items	Median nerve injury (n = 115)				Ulnar nerve injury (n = 94)				Radial nerve injury (n = 72)			
	**Preoperative** **n = 50**	**3 months n = 36**	**12 months n = 59**	**P-value**	**Preoperative** **n = 42**	**3 months n = 25**	**12 months n = 52**	**P-value**	**Preoperative** **n = 32**	**3 months n = 29**	**12 months n = 39**	**P-value**
Cold sensitivity	1 [0–44]a	67 [7–90]	70 [40–90]c	**<0.001**	15 [0–50]^a^	60[30–85]	73 [30–99]^c^	**<0.001**	1 [0–20]	20 [0–70]	20 [0–71]	0.061
Pain at rest	1 [0–20]	10 [1–38]	10 [1–30]	0.097	1 [0–30]	20 [2–40]	20 [2–50]	0.064	1 [0–25]	2 [0–10]	10 [0–20]	0.946
Pain on motion without load	1 [0–30]	20 [2–40]	13 [0–40]	0.121	6 [0–50]	20 [3–45]	20 [3–50]	0.156	8 [0–53]	20 [3–44]	10 [0–20]	0.252
Pain on load	1 [0–60]^a^	43[23–63]	36 [20–60]^c^	**0.022**	1 [0–74]	40 [15–61]	40 [13–70]	0.056	15 [0–70]	20 [11–72]	20 [0–50]c	0.381
Stiffness	2 [0–50]^a^	30 [3–60]	34 [20–50]^c^	**0.009**	10 [0–50]^a^	50 [25–74]	50 [30–70]	**0.005**	4 [0–45]	30 [5–55]	20 [0–40]	0.166
Weakness	2 [0–60]^a^	56[40–70]^b^	31 [20–60]^c^	**<0.001**	10 [0–70]^a^	50 [35–70]	67 [40–80]	**0.001**	9 [0–52]a	50 [15–70]b	20 [10–40]c	**0.011**
Numbness, tingling in fingers	15[0–60]^a^	57[30–80]	60 [30–80]^c^	**<0.001**	6 [0–70]^a^	57 [30–76]	70 [40–89]	**<0.001**	10 [0–38]	30 [8–61]	20 [10–60]	0.231
Ability to perform daily activities	1 [0–50]^a^	40[20–64]	30 [10–50]^c^	**0.015**	1 [0–70]	40 [15–55]	42 [30–70]	0.065	17 [0–50]	30 [7–55]	10 [0–31]c	0.227

Data are presented as median [IQR; interquartile range]. Significance testing with Kruskal-Wallis and post-hoc Mann-Whitney U-test were used.

*Bold values indicate p-value <0.05.

*a* indicates p<0.05 between preoperative and 3 months postoperative values

*b* indicates p < 0.05 between 3 months postoperative and 12 months postoperative

*c* indicates p <0.05 between preoperative and 12 months postoperative.

Individuals with a median or an ulnar nerve injury, but not individuals with a radial nerve injury, reported an increase in stiffness at three months postoperatively; which persisted at 12 months postoperatively (p<0.009 and p = 0.005, respectively). A significant increase in weakness was seen in regard to all three nerve trunk injuries at three months postoperatively, with a further increase in weakness at 12 months postoperatively for the individuals with a median or a radial nerve injury, whereas symptoms persisted at 12 months for the group with an ulnar nerve injury (p<0.001, p = 0.011 and p = 0.001, respectively).

Numbness/tingling in fingers was also reported for the median and the ulnar nerve injury group, but not for the radial nerve injury group, at three months postoperatively, which persisted at 12 months postoperatively (p<0.001 and p<0.001, respectively).

A significant decrease in the ability to perform daily activities was seen for the individuals with a median nerve injury, but not for the other two nerve injury groups, at three months postoperatively that persisted at 12 months postoperatively (p<0.015) (Table 2, Fig 2). Overall, a similar pattern was seen in cold sensitivity and functional outcome for individuals with a median and an ulnar nerve injury at 12 months, while individuals with a radial nerve injury, overall, had milder cold sensitivity and better functional outcome. When comparing only the median and ulnar nerves, significant differences were seen in stiffness (p = 0.019), weakness (<0.001) and ability to perform daily activities (p = 0.003) at 12 months postoperatively, indicating the ulnar nerve as the nerve trunk injury with worst outcome.

### Sex differences

No difference in cold sensitivity scores was found between men and women with a median (70 [50–90] and 56 [12–83]; p = 0.134), an ulnar (70 [16–100] and 82 [30–98]; p = 0.553) or a radial (20 [0–71] and 20 [0–98]; p = 0.921) nerve injury at 12 months postoperatively.

A statistically significant difference was found in stiffness between men and women with a median nerve injury (40 [20–60] and 30 [5–41]; p = 0.034) at 12 months postoperatively, but none of the other variables differed for the individuals with median, ulnar or radial nerve injuries. Furthermore, no difference in QuickDASH scores 12 months postoperatively between men and women with median (30 [23–41] and 28 [15–44]; p = 0.580), ulnar (34 [24–65] and 34 [16–60]; p = 0.716), or radial (17 [6–36] and 14 [4–45]; p = 0.966) nerve injuries were found. Concomitant injuries were equally common among women and men regardless of injured nerve trunk.

### Correlations between QuickDASH, cold sensitivity and related symptoms

**Fig 3:**
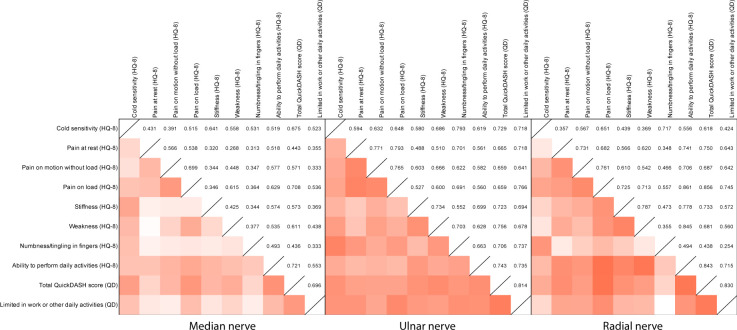
Correlation between cold sensitivity and other HQ-8 symptoms for a median, ulnar and radial nerve injury. Data are rho-values (Spearman rank test). Rho-values <0.3: Weak correlation, Rho-values 0.3–0.7: Moderate correlation and >0.7: Strong correlation. All p-values <0.05.

We found a strong correlation (Rho value >0.7) between cold sensitivity and tingling/numbness at 12 months for the radial and ulnar nerve. For ulnar nerve injuries there was also a strong correlation between cold sensitivity and limitations in work and other daily activities as well as total QuickDASH score at 12 months (Fig 3). Cold sensitivity correlated moderately (Rho value >0.5–0.7) with all other evaluated symptoms in the HQ-8, and with limitations in work and other daily activities for all three nerve trunk injuries (Fig 3).

For median nerve injuries, the total QuickDASH score at 12 months correlated strongly with pain on load and the ability to perform daily activities. For ulnar nerve injuries, the total QuickDASH score at 12 months correlated strongly with stiffness, weakness, numbness and the ability to perform daily activities. For radial nerve injuries, we found strong correlations between the total QuickDASH score at 12 months and pain at rest, pain on load, stiffness and ability to perform daily activities. Overall, in radial and ulnar nerve injuries the evaluated symptoms in HQ-8 correlated more often strongly to QuickDASH in contrast to the median nerve injuries, where correlations were more moderate (Fig 3).

### Predictors for cold sensitivity

The multiple regression analysis was performed for the median, ulnar and radial nerve injuries, respectively. No significant effect in predicting cold sensitivity at 12 months was found for the individuals with median (adjusted for age, sex, diabetes and concomitant flexor tendon injury), ulnar (adjusted for age, sex, diabetes and concomitant flexor tendon injury and vascular injury) or radial (adjusted for age, sex, diabetes and concomitant extensor tendon injury) nerve injuries.

### Cold sensitivity, QuickDASH and concomitant injuries

Individuals with a median nerve injury had significantly higher QuickDASH scores at three months postoperatively compared to preoperatively (p = 0.012) that remained significantly higher at 12 months postoperatively (p = 0.019), which was not observed for individuals with an ulnar or a radial nerve injury. Individuals with a radial nerve injury had significantly lower QuickDASH scores at 12 months postoperatively compared to individuals with a median or an ulnar nerve injury (Table 3). With all nerve trunk injuries combined, we found no differences between the group with severe cold sensitivity compared to the group with mild cold sensitivity in QuickDASH score at 3 months postoperatively (p = 0.968).

**Table 3:** QuickDASH score for individuals treated with repair or reconstruction of an injured major nerve trunk injury in the upper extremity.

QuickDASH score	Median nerve (n = 115)	Ulnar nerve (n = 94)	Radial nerve (n = 72)	P-value
Preoperative QuickDASH score	7 [0–47]^**a**^ (n = 50)	26 [0–72] (n = 42)	29 [0–55] (n = 31)	0.406
QuickDASH score at three months	38 [22–57] (n = 34)	43 [23–64] (n = 23)	27 [11–42] (n = 30)	0.096
QuickDASH score at 12 months	30 [21–41]^**b**^ (n = 56)	34 [22–64]^**c**^ (n = 50)	17 [6–34] (n = 36)	**<0.001**
P-value	**0.019**	0.423	**<0.001**	

Values are presented as median [IQR; interquartile range]. Significance testing with Kruskal-Wallis Wallis and post-hoc Mann-Whitney U-test were used.

*Bold values indicate p <0.05 and are considered statistically significant.

*a* indicates p<0.05 between preoperative QuickDASH score and QuickDASH score at three months for the median nerve

*b* indicates p < 0.05 between QuickDASH score at 12 months for the median and radial nerve values

*c* indicates p < 0.05 between QuickDASH score at 12 months for the median and radial nerve values.

The univariate general linear regression analysis showed that the total QuickDASH score for the individuals with a median nerve injury at 12 months (adjusted for age, sex, diabetes and flexor tendon injury) was higher for the group with severe cold sensitivity than for those with mild cold sensitivity; with an adjusted mean difference of 24.8 points (95% CI 12.2–37.4; p<0.001).

The total QuickDASH score for the individuals with an ulnar nerve injury at 12 months (adjusted for age, sex, diabetes, flexor tendon injury and vascular injury) was higher for the group with severe cold sensitivity than for those with mild cold sensitivity; with an adjusted mean difference of 36.5 points (95% CI 23–49.9; p<0.001).

The total QuickDASH score for the individuals with a radial nerve injury at 12 months (adjusted for age, sex, diabetes and extensor tendon injury) was higher for the group with severe cold sensitivity than for those with mild cold sensitivity; with an adjusted mean difference of 30.3 points (95% CI 15.1–45.5; p<0.001).

### Responders and non-responders

Of the 281 individuals included in the study, 123/281 (44%) responded to the HAKIR questionnaire 8 (HQ-8) preoperatively, 87/281 (31%) at three months, and 142/281 (51%) responded at 12 months postoperatively. There were no differences in age or sex when comparing the included individuals to non-responders (i.e. individuals who had not filled in any HQ-8 at any time-point).

## Discussion

Cold sensitivity and numbness/tingling in fingers were the most prominent self-reported symptoms after an injured and repaired or reconstructed median or ulnar nerve with or without concomitant injuries up to 12 months after surgery. Cold sensitivity was also associated with self-reported disability at 12 months after repair or reconstruction of an injured nerve trunk, as demonstrated by higher QuickDASH scores with a more severe cold sensitivity category for all three nerve trunk injuries, indicating that cold sensitivity may have a association to disability after a surgically treated major nerve trunk injury in the upper extremity. Furthermore, there were no differences in cold sensitivity or in QuickDASH score at 12 months between surgical methods, i.e. direct nerve repair or nerve reconstruction with nerve autografts, indicating that it is the nerve injury that is relevant and not the surgical technique. Earlier studies support this finding [26–29]. However, we did not have information on the autograft length, where longer autografts are known to have worse outcome, and there were only few patients treated with nerve autografting, so further studies are needed to confirm this notion [30,31]. There was also an impaired ability to perform daily activities among the individuals with a median or an ulnar nerve injury, while individuals with a radial nerve injury had less problems, probably due to the fact that most of the injuries to the radial nerve affected its superficial sensory branch, since we only included injuries of the forearm and wrist. Interestingly, individuals with an ulnar nerve injury exhibited more problems to perform daily activities than those with a median nerve injury. This may be caused by the important motor function of the ulnar nerve driven by the intrinsic muscles of the hand and possibly the large effect of misdirection of regenerating axons when repairing the nerve, even if misdirection may be more relevant in regeneration of sensory axons [32,33]. In accordance, weakness was rated highly by individuals with an ulnar nerve injury and weakness correlated stronger to the other evaluated symptoms in the HQ-8 and QuickDASH compared to the median nerve injuries. The median nerve has previously been described as having better motor recovery than the ulnar nerve [30,34,35]. This is supported by the findings of Bruyns et al., who found that individuals with isolated median nerve injuries returned to work within one year more frequently than patients with isolated ulnar nerve injuries (80% vs 59%, respectively) [36]. Also, Ruijs et al., found that an ulnar nerve injury have 71% lower chance of motor recovery than a median nerve injury [37]. The reason why individuals with a radial nerve injury reported significant weakness at 12 months postoperatively is obscure. In one study, investigating the results after a radial nerve injury repair, neuroma formation in the forearm of the superficial radial nerve branch was common [38]. We speculate that the pain associated with a neuroma can lead to secondary weakness. However, individuals with a radial nerve injury did not report any other prominent symptoms in contrast to individuals with a median or an ulnar nerve injury. The radial nerve innervates foremost the dorso-radial area of the hand and forearm. It is possible that loss of sensation on the dorsal side of the hand is not perceived as problematic as volar side sensory impairment. The volar side includes the fingertips, and is far more exposed than the area innervated by the radial nerve. Also, two point discrimination is naturally much more discriminate on the volar compared to the dorsal aspect of the hand (mean 3.8–5.9 mm; and 11.1–12.2 mm, respectively) [39].

Neither age, nor sex, affected QuickDASH scores at 12 months, which also has been reported among individuals with a single digital nerve injury [20]. This is in contrast to previous studies, where lower age has been reported as a significant predictor of successful motor recovery [32,37]. However, the present individuals with a major nerve trunk injury showed 4–16 points higher QuickDASH score at 12 months postoperatively when the self-reported cold sensitivity was judged as severe (i.e. >70) compared to individuals with a single digital nerve injury [20], indicating the impact of particularly the median and ulnar nerves on hand function. The present QuickDASH scores for individuals with a median and an ulnar nerve injury (i.e. 30–34) are in agreement with the results of a previous study of individuals with similar injuries (i.e. 29–31) [40].

No difference in cold sensitivity symptoms was found between men and women, nor among any of the self-reported disability symptoms, except for stiffness in men with a median nerve injury at 12 months postoperatively. However, it is important to note that stiffness could also depend on other patient factors, such as a previous injury or a joint disease. Previous studies, including various diagnoses, show commonly a sex difference regarding QuickDASH score. We wonder if the injury area, with the need for more extensive injury to reach the major nerve trunks, plays a role. Furthermore, one may speculate if the individuals, women or men, that get injured perhaps verge to have more dangerous jobs or hobbies and in that sense are not directly comparable to the general population that suffers from for example a digital nerve injury after a smaller knife cut to a finger or the often slightly older population that suffer from carpal tunnel syndrome [19].

We found no differences in occurrence of concomitant injuries between men and women, which is in contrast to individuals with a single injury to a digital nerve, where men tend to suffer from more severe and multiple injuries [20]. This could be explained by the position of the major nerve trunks, which commonly lie deep to the flexor tendons on the volar aspect, depending on the level, where an injury to the median or the ulnar nerves needs to be deep enough to perch the nerve and therefore often involves a more extensive and complex injury regardless of sex. Among the individuals in our study, flexor tendon injury was by far the most common concomitant injury for both a median or an ulnar nerve trunk injury due to the close association with these structures on the volar side of the forearm. Interestingly, no difference was seen in cold sensitivity scores, nor QuickDASH scores, at 12 months between individuals with or without concomitant flexor tendon injury. Furthermore, individuals with a median or an ulnar nerve injury, who reported severe cold sensitivity at 12 months, did not more commonly have concomitant flexor tendon injury, which is in contrast to individuals with a digital nerve injury [20]. The location of the injury at the wrist/forearm level might play a role concerning the start of rehabilitation, since such a program may be initiated earlier concerning mobilisation of the fingers and the postoperative range of motion is not affected as much as compared to an injury at the digital level. The loss of both sensation and motor function might play a role in burying the symptoms of the tendon injury. A more extensive injury, where the motor function is not expected to return until after the first 3 months postoperatively and healing of concomitant injury of a tendon is possibly achieved before the individual can activate it, might play a role in the perceived disability and cold sensitivity.

Extensor tendon injury was the most common concomitant injury among the individuals with a radial nerve trunk injury due to the close relation to the radial nerve on the dorso-radial aspect of the forearm. Again, no differences were seen in cold sensitivity scores nor QuickDASH scores at 12 months between individuals with or without concomitant extensor tendon injury. Furthermore, individuals with a median and an ulnar nerve injury, reporting severe cold sensitivity at 12 months, did not more commonly have concomitant flexor tendon injury, which is in contrast to individuals with a digital nerve injury [20]. A vascular injury has long been associated with cold sensitivity [11,41], and such an injury was the second most common concomitant injury with an ulnar nerve trunk injury, which is understandable due to the close relation of the nerve to the ulnar artery buried under the flexor carpi ulnaris. Yet, no difference in cold sensitivity or QuickDASH score was seen at 12 months postoperatively for individuals with or without concomitant vascular injury with an ulnar nerve trunk injury, which may be explained by the fact that the remaining cold sensitivity after such a nerve injury predominate over any impact of a vascular injury. Altogether, this indicates that the prolonged disability after a repaired or reconstructed median or an ulnar nerve injury at 12 months may be related to presence of cold sensitivity and not the concomitant injuries.

The season during which the individuals were operated did not have any effect on the cold sensitivity scores. The individuals in our study were treated at hospitals and units throughout Sweden; thus, with very different climate during the various time periods. Although a seasonal variation in cold sensitivity has been reported after repair of various nerve injuries in a smaller study [9], other previous studies on cold sensitivity and its severity support our finding that cold sensitivity results are very similar between regions with different climate and temperatures [20,23,42].

The limitations of the HAKIR registry are that no detailed information is provided considering the details of the mechanism of injury, how long after the trauma the surgery was performed or if the individuals had previous injuries on the same arm. No information on the general health of the individuals nor their habits, e.g. any use of tobacco, which has previously been associated with cold sensitivity [7], was available. We also had no information regarding BMI, where overweight (BMI 25–30) previously has been associated with lower reported frequency of cold sensitivity, or other underlying diseases than diabetes, which also may influence the presence of cold sensitivity [4,43,44]. Furthermore, the individual´s motivation to complete physical hand therapy, their cognitive capacity and participation is unknown [16,45]. Information on sensory recovery (2-PD) was not included, an important factor that can be correlated to neuropathic pain indicating that the better the sensory recovery is in a patient, the less likely they are to suffer from cold sensitivity and neuropathic pain [10]. In regard to the questionnaires, individuals, in some cases, report high scores preoperatively, especially individuals with a radial nerve injury. If that is due to them already having a disability, e.g. due to a previous injury or if they have misunderstood and reported their status in accordance to after the injury is unknown. In this case the calculated severity of cold sensitivity and other factors is only underestimated. The rate of same responders to all three questionnaires is also a limitation in the present study. Thus, paired analyses were not applicable for significance testing over time. However, the response rate corresponds well to previously published studies [19,20], and the drop-out analysis showed no significant difference in the population of responders compared to the non-responders. The risk for sampling bias was considered low in accordance to the findings of Stirling et al that no significant difference in mean postoperative QuickDASH score was observed between responders and predicted scores of non-responders, even with the non-responders group differing in age, comorbidity, employment status and having wores preoperative QuickDASH scores [46]. The strengths of this study are the high number of included individuals compared to previously published studies on the subject, the nationwide inclusion of patients through a national registry and the use of two PROMs to evaluate symptoms and disability.

## Conclusion

Cold sensitivity after surgery for a major nerve trunk injury in the upper extremity up to 12 months postsurgery can be substantial and lead to disability and decreased ability to perform daily activities. An ulnar nerve injury appears to have the worst outcome of the three major nerve trunk injuries. Cold sensitivity is an important outcome. We suggest that it should be evaluated in addition to sensory and motor function and pain in individuals after a repaired or reconstructed major nerve trunk injury in the upper extremity.
